# Challenges, Perceptions, Training and Needs of Primary Care Nurses in the Management of Patients with Percutaneous Endoscopic Gastrostomy: A Cross-Sectional Study in Southern Spain

**DOI:** 10.3390/healthcare13212786

**Published:** 2025-11-03

**Authors:** María de los Santos Oñate-Tenorio, Nuria Trujillo-Garrido, María de los Ángeles Bernal-Jiménez, María José Santi-Cano

**Affiliations:** 1 Emergency Clinical Management Unit, Andalusian Health Service, 11630 Cadiz, Spain; mariasantos.onate@uca.es; 2 Research Group on Nutrition, Molecular, Pathophysiological and Social Issues, University of Cádiz, 11009 Cadiz, Spain; mariangeles.bernaljimenez@gmail.com (M.d.l.Á.B.-J.); mariajose.santi@uca.es (M.J.S.-C.); 3 Nursing and Physiotherapy Department, Faculty of Nursing and Physiotherapy, University of Cádiz, 11009 Cadiz, Spain; 4 Institute of Biomedical Research and Innovation of Cádiz (INiBICA), 11009 Cadiz, Spain; 5 Nursing and Physiotherapy Department, Faculty of Nursing, University of Cádiz, 11207 Cadiz, Spain

**Keywords:** gastrostomy, primary care nursing, home care services, training requirements

## Abstract

**Background/Objectives**: The use of percutaneous endoscopic gastrostomy (PEG) offers numerous benefits but is also associated with complications that require specialised management. However, evidence regarding the management of these patients in primary care, both in Spain and internationally, remains limited. The aim of this study was to analyse primary care nurses’ perceptions of their level of knowledge and their needs related to the management of patients with PEG tubes. **Methods**: A descriptive cross-sectional study was conducted, using an online questionnaire addressed to primary care nurses in Andalusia, Spain. Demographic data, perceived level of knowledge, and perceptions regarding PEG management were collected. Statistical analysis included chi-square and Fisher’s exact tests, as well as a multivariable logistic regression model. **Results**: A total of 121 nurses participated, where 92.4% had cared for PEG patients and 68.9% had managed complications. Within the total group, older nurses and those with more years of professional experience demonstrated significantly greater knowledge in managing these complications (*p* < 0.05). However, only 38.5% had received specialised clinical training, and 98.3% expressed the need for continuing professional development. The lack of up-to-date protocols was a recurrent finding, with many nurses relying on referrals to specialist colleagues. **Conclusions**: Primary care nurses frequently manage PEG-related complications but lack systematic access to evidence-based guidelines and training. This gap underscores the need for structured educational programmes such as practical seminars, simulation-based online modules, regular clinical sessions led by specialist nurses, and clear visual protocols for identifying and managing common complications.

## 1. Introduction

Percutaneous Endoscopic Gastrostomy (PEG) is a method of feeding through the endoscopic and percutaneous placement of a tube into the stomach by creating a gastrocutaneous fistula. This technique was first described in the 1980s as a less invasive alternative to surgical gastrostomy [1,2]. PEG is indicated when enteral nutrition is required for more than four weeks, aiming to prevent complications associated with the use of a nasogastric tube [3]. It is a long-term home enteral nutrition technique whose use has increased in recent years due to factors such as population ageing and advances in the management of certain digestive surgical procedures [4].

The use of PEG offers advantages over other types of enteral nutrition in the medium and long-term but can also result in various complications [5]. While the external tube of the PEG can remain in place for six months and the internal tubes for one to two years, PEG patients may experience complications during these periods [6,7]. Early complications, occurring before the formation of a stable stoma (usually within four weeks), include PEG tube dislodgement, intraperitoneal leakage, pneumoperitoneum, infection around the fistula, skin ulcers, and necrotising fasciitis. Late complications, which occur after the maturation of the PEG tract, commonly involve accidental tube dislodgement and obstruction, as well as other issues such as buried bumper syndrome, tube rupture, granuloma formation, and fistulas [3].

The prevalence of each complication varies across publications, although accidental tube dislodgement, obstruction, and infection in the stoma area are among the most commonly reported issues [8,9,10]. For these reasons, patients or their caregivers often seek the assistance of primary care nurses, either due to complications or for tube replacement [11,12]. Therefore, caring for patients with PEG involves significant responsibilities, necessitating the availability of guidelines, protocols, and educational programmes to train caregivers.

However, the current level of evidence regarding the care of these patients in our setting is limited. The European Society for Clinical Nutrition and Metabolism (ESPEN) published guidelines on PEG management in 2005 [13], and no new European guidelines have been issued since then. More recently, the Korean Society of Endoscopy released an updated clinical practice guideline [3], concluding that most existing recommendations remain of low strength and are primarily based on expert consensus. This underscores the urgent need for studies that reflect the realities of different healthcare systems and national contexts. In Spain, despite the growing use of PEG as a long-term home enteral nutrition method, to our knowledge, there are no national nursing guidelines or empirical studies examining how these patients are managed in primary care. Given that in Spain, primary care nurses are the professionals primarily responsible for providing home-based care to PEG patients—including follow-up, management of minor complications, and patient and caregiver education—this study focused on their perceptions and training needs regarding PEG management.

Therefore, the aim of this study was to analyse primary care nurses’ perceptions of their level of knowledge and their needs related to the management of patients with PEG tubes, in order to promote evidence-based care aligned with healthcare demands and to provide context-specific insights that may inform future clinical and educational strategies.

## 2. Materials and Methods

### 2.1. Design

We conducted a cross-sectional descriptive study using online questionnaires between March and May 2025. The study collected responses from primary care nurses in Andalusia, Spain. A bibliographic search confirmed that no existing instrument measured the primary care nurses’ perceptions of their own knowledge and training needs regarding PEG tube management. Subsequently, the research team designed an ad hoc questionnaire specifically developed for this study. The initial version contained 11 items formulated after exploring the concept of PEG management in nursing practice and identifying key thematic areas. This preliminary version was reviewed by two expert primary care nurses and subsequently by four researchers who acted as judges. Following their feedback, the instrument was expanded to 14 items, which constituted the final version used for data collection. To ensure clarity and feasibility, the questionnaire was pilot-tested with eight primary care nurses to ensure clarity and feasibility before data collection.

The final questionnaire comprised three sections: (a) Demographic and professional background information, (b) Categorical response questions on knowledge and experiences related to PEG tube replacement and complications, and (c) An open-ended question about personal perceptions regarding the level of knowledge of primary care nurses in managing patients with PEG tubes. The items included in the questionnaire are listed in Table 1.

### 2.2. Study Setting and Sampling

We conducted this study in the region of Andalusia, Spain, targeting nurses working in primary care within the Andalusian Health Service (Servicio Andaluz de Salud, SAS). Primary Healthcare Services are organised into Primary Care Districts, which comprise Primary Healthcare (PHC) and Primary Care Emergency Service (PCES). The latter provide urgent medical care outside regular health centre hours. Primary care nurses were invited to participate in the study via email. Each email contained a unique link to prevent reuse and duplicate submissions. A total of 505 email invitations were sent, resulting in 121 valid responses (response rate: approximately 24%). Participation was voluntary; therefore, a voluntary response sampling approach was used. The sample size was determined based on Hair and Anderson’s recommendation of including at least 100 participants to ensure adequate statistical validity in multivariate analyses [14].

### 2.3. Variables

The questionnaire included sociodemographic and professional variables, as well as items related to knowledge, experience, and perceptions regarding PEG tube management.

Sociodemographic and professional variables comprised age (categorized as follows: 26–35 years; 36–45 years; 46–555 years; 56–65 years), sex (female or male), and professional experience (categorized as follows: 3–10 years; 11–20 years; 21–30 years; more than 30 years), with less than three years of professional experience considered an exclusion criterion. The health service where the participants worked (Primary Healthcare or Primary Care Emergency Service) and workplace location (urban: more than 50,000 inhabitants; semi-rural: between 5000 and 49,999 inhabitants; or rural: up to 4999 inhabitants) were also recorded.

Academic level referred to the highest qualification obtained (nursing diploma, bachelor’s degree, master’s degree, or PhD). This variable was distinct from the Specialist in Family and Community Nursing (S-FCN) category, which represents a postgraduate clinical specialization.

Knowledge, experience, and training variables addressed the participants’ exposure to PEG patients, management of complications (e.g., accidental dislodgement, obstruction, infection), participation in PEG-related procedures, and prior training on PEG care.

Perception variables included the perceived need for additional training and the open-ended question about nurses’ knowledge and care of PEG patients. A full list of questionnaire items is provided in Table 1.

### 2.4. Data Collection Data Analysis

Data were collected using Google Forms and exported to Microsoft Excel, where completeness was verified, and responses with missing data were cleaned. Chi-square and Fisher’s exact tests were employed, with a significance level set at *p* < 0.05. In addition, a multivariable logistic regression analysis was performed to examine the association between independent variables (age, educational level, being a Family and Community Nursing specialist, years of professional experience, and type of health service where participants worked) and the responses to each questionnaire item. Odds ratios (OR) with 95% confidence intervals (CI) were calculated. Data analysis was conducted using SPSS software, version 24. A qualitative analysis of the responses to the open-ended question was performed through coding, categorisation, and thematic analysis of their content.

### 2.5. Ethical Considerations

The study was conducted in compliance with the ethical principles outlined in the Declaration of Helsinki, issued in Fortaleza (Brazil) in October 2013, the Spanish Biomedical Research Act 14/2007, and the European General Data Protection Regulation. Participants were informed about the study’s objectives, the anonymous and voluntary nature of the questionnaire, and their right to withdraw at any time and request the removal of their data from the study. The data collected was exclusively used for the stated research purposes. Participation required prior consent, without which access to the questionnaire was not permitted.

## 3. Results

A total of 121 primary care nurses participated in the study. The sociodemographic characteristics of the participants are detailed in Table 2. Most participants were women (67.2%) aged between 46 and 65 years (70%), with a qualification level of nursing diploma/degree (60.7%) or specialising in Family and Community Nursing (S-FCN) (24.6%). Additionally, 45.9% had over 30 years of professional experience. In terms of work setting, 91.8% of the nurses were employed in Primary Healthcare centres and 7.4% in Primary Care Emergency Services, providing care mainly in urban (59.0%) or intermediate (29.5%) areas (Table 2). As for the questionnaire results, no differences were observed between women and men or among the different population settings where nurses worked (urban, intermediate, or rural). However, significant differences were identified among the different age groups, years of professional experience, educational level, and type of health service (primary healthcare or Primary Care Emergency Service), as shown in Table 3, Table 4, Table 5 and Table 6.

Nearly all nurses (92.4%) reported having cared for a patient with a PEG tube, with no differences observed across age groups, professional experience, education level, or healthcare service. At the time of the study, 44.5% of the participating nurses were actively caring for PEG patients. This outcome was directly associated with years of professional experience (*p* = 0.030) and educational level, with specialist nurses providing care at a significantly higher rate (76.7%) compared to other qualification levels (*p* < 0.001) (Table 3).

A total of 68.9% of the nurses had been called upon to address a PEG tube complication, with no significant differences based on age, experience, education, or healthcare service. The most frequently reported complication was accidental dislodgement (51.0%), followed by obstruction (28.4%) and infection (20.6%), with significant differences observed by age group. Among younger nurses, obstruction was the primary complication, whereas older nurses most commonly encountered accidental dislodgement (*p* = 0.031) (Table 3).

**Table 3:** Results for Questions 1 to 4.

						Total	*p*
Q1	Age (years) % (*n*)	(26–35)	(36–45)	(46–55)	(56–65)		0.152
		30.8 (4)	33.3 (7)	41.7 (15)	57.1 (28)	45.4 (54)	
	Professional experience (Years) % (*n*)	(3–10)	(11–20)	(21–30)	(>30)		0.030
		10.0 (1)	27.8 (5)	48.6 (17)	53.6 (30)	44.5 (53)	
	Education level % (*n*)	Nursing degree	Master’s degree	S-FCN	PhD (HS)		<0.001
		32.4 (24)	36.4 (4)	76.7 (23)	50.0 (3)	44.6 (54)	
	Healthcare service % (*n*)	PHC	PCES				1.000
		44.6 (50)	44.4 (4)			44.6 (54)	
Q2	Age (years) % (*n*)	(26–35)	(36–45)	(46–55)	(56–65)		0.486
		92.3 (12)	85.0 (17)	91.7 (33)	95.9 (47)	92.4 (109)	
	Professional experience (Years) % (*n*)	(3–10)	(11–20)	(21–30)	(>30)		0.471
		90.0 (9)	88.9 (16)	88.2 (30)	96.4 (54)	92.4 (109)	
	Education level % (*n*)	Nursing degree	Master’s degree	S-FCN	PhD (HS)		0.268
		89.0 (65)	90.9 (10)	100 (30)	83.3 (5)	91.7 (110)	
	Healthcare service % (*n*)	PHC	PCES				0.556
		91.9 (102)	88.9 (8)			91.7 (110)	
Q3	Age (years) % (*n*)	(26–35)	(36–45)	(46–55)	(56–65)		0.889
		69.2 (9)	61.9 (13)	69.4 (25)	71.4 (35)	68.9 (82)	
	Professional experience (Years) % (*n*)	(3–10)	(11–20)	(21–30)	(>30)		0.884
		60.0 (6)	66.7 (12)	71.4 (25)	71.4 (40)	69.7 (83)	
	Education level %(*n*)	Nursing degree	Master’s degree	S-FCN	PhD (HS)		0.098
		62.2 (46)	81.8 (9)	83.3 (25)	50.0 (3)	68.6 (83)	
	Healthcare service % (*n*)	PHC	PCES				0.718
		67.9 (76)	77.8 (7)			68.6 (83)	
Q4	Age (years) % (*n*)	(26–35)	(36–45)	(46–55)	(56–65)		0.031
	Dislodgement	9.1 (1)	52.9 (9)	56.3 (18)	57.1 (24)	51.0 (52)	
	Obstruction	72.7 (8)	23.5 (4)	18.8 (6)	26.2 (11)	28.4 (29)	
	Infection	18.2 (2)	23.5 (4)	25.0 (8)	16.7 (7)	20.6 (21)	
	Professional experience (Years) % (*n*)	(3–10)	(11–20)	(21–30)	(>30)		0.200
	Dislodgement	22.2 (2)	33.3 (5)	64.5 (20)	52.1 (25)	50.5 (52)	
	Obstruction	55.6 (5)	40.0 (6)	16.1 (5)	27.1 (13)	28.2 (29)	
	Infection	22.2 (2)	26.7 (4)	19.4 (6)	20.8 (10)	21.4 (22)	
	Education level % (*n*)	Nursing degree	Master’s degree	S-FCN	PhD (HS)		0.212
	Dislodgement	60.7 (37)	22.2 (2)	41.4 (12)	20.0 (1)	50.0 (52)	
	Obstruction	23.0 (14)	44.4 (4)	34.5 (10)	40.0 (2)	28.8 (30)	
	Infection	16.4 (10)	33.3 (3)	24.1 (7)	40.0 (2)	21.2 (22)	
	Healthcare service % (*n*)	PHC	PCES				0.288
	Dislodgement	52.1 (50)	25.0 (2)			50.0 (52)	
	Obstruction	27.1 (26)	50.0 (4)			28.8 (30)	
	Infection	20.8 (20)	25.0 (2)			21.2 (22)	

Q1: Do you currently care for any patients with PEG tubes? Q2: Have you ever cared for a patient with a PEG tube? Q3: Have you been called upon to address a PEG tube complication? Q4: Which of the following complications have you most frequently encountered? (Accidental dislodgement, obstruction, infection). S-FCN: Specialist in Family and Community Nursing; PhD (HS): Doctorate in Health Sciences; PHC: Primary Healthcare; PCES: Primary Care Emergency Service. Associations between categorical variables were analysed using the Chi-square test or Fisher’s exact test when the expected frequencies were <5. Statistical significance was set at *p* < 0.05.

A total of 57.0% of nurses reported knowing what to do in the event of accidental PEG tube dislodgement, with significant differences observed based on their work setting: 59.8% in PHC compared to 22.2% in PCES (*p* = 0.038). Following an accidental dislodgement, 48.7% of nurses had reinserted the tube, primarily those with greater age (*p* < 0.001) and professional experience (*p* < 0.001). In contrast, only 10% of nurses with 3–10 years of experience and 16% of those with 11–20 years of experience had reinserted the tube (Table 4).

In cases of PEG tube obstruction, 72.9% of nurses reported knowing the appropriate course of action, with knowledge being significantly more prevalent among older nurses (*p* = 0.012) and those with greater professional experience (*p* = 0.003). Additionally, 31.1% had replaced a PEG tube due to obstruction at some point, with no significant differences based on age, experience, education, or healthcare service (Table 4).

A total of 53.8% of nurses had replaced a PEG tube at its scheduled renewal date, with this practice being more common among older nurses (*p* = 0.003) and those with greater professional experience (*p* < 0.001) (Table 4).

Logistic regression analyses revealed that working in Primary Healthcare was associated with a higher likelihood of knowing what to do in the event of accidental PEG tube dislodgement (Q5; OR = 0.19 for PCES areas). In addition, greater professional experience significantly increased the likelihood of having reinserted a dislodged tube (Q6; OR = 8.76), having replaced a tube due to obstruction (Q8; OR = 3.41), and having performed a scheduled tube replacement (Q9; OR = 6.15).

**Table 4:** Responses to Questions 5 to 9.

						Total	*p*	OR (95% CI) *p*
Q5	Age (years) % (*n*)	(26–35)	(36–45)	(46–55)	(56–65)		0.232	
		38.5 (5)	47.6 (10)	66.7 (24)	61.2 (30)	58.0 (69)		
	Professional experience (Years) % (*n*)	(3–10)	(11–20)	(21–30)	(>30)		0.543	
		50.0 (5)	44.4 (8)	60.0 (21)	62.5 (35)	58.0 (69)		
	Education level % (*n*)	Nursing degree	Master’s degree	S-FCN	PhD (HS)		0.520	
		59.5 (44)	45.5 (5)	60.0 (18)	33.3 (2)	57.0 (69)		
	Healthcare service % (*n*)	PHC	PCES				0.038	0.19 (0.38–0.966) *p* = 0.045
		59.8 (67)	22.2 (2)			57.0 (69)		
Q6	Age (years) % (*n*)	(26–35)	(36–45)	(46–55)	(56–65)		0.001	
		15.4 (2)	23.8 (5)	55.6 (20)	63.3 (31)	48.7 (58)		
	Professional experience (Years) % (*n*)	(3–10)	(11–20)	(21–30)	(>30)		<0.001	8.75 (2.80–27.33) *p* = 0.000
		10.0 (1)	16.7 (3)	57.1 (20)	60.7 (34)	48.7 (58)		
	Education level % (*n*)	Nursing degree	Master’s degree	S-FCN	PhD (HS)		0.347	
		47.3 (35)	45.5 (5)	56.7 (17)	16.7 (1)	47.9 (58)		
	Healthcare service % (*n*)	PHC	PCES				0.494	
		49.1 (55)	33.3 (3)			47.9 (58)		
Q7	Age (years) % (*n*)	(26–35)	(36–45)	(46–55)	(56–65)		0.012	
		69.2 (9)	47.6 (10)	71.4 (25)	85.7 (42)	72.9 (86)		
	Professional experience (Years) % (*n*)	(3–10)	(11–20)	(21–30)	(>30)		0.003	
		60.0 (6)	55.6 (10)	60.0 (21)	89.1 (49)	72.9 (86)		
	Education level % (*n*)	Nursing degree	Master’s degree	S-FCN	PhD (HS)		0.243	
		67.1 (49)	72.7 (8)	86.7 (26)	66.7 (4)	72.5 (87)		
	Healthcare service % (*n*)	PHC	PCES				0.257	
		73.9 (82)	55.6 (5)			72.5 (87)		
Q8	Age (years) % (*n*)	(26–35)	(36–45)	(46–55)	(56–65)		0.470	
		23.1 (3)	19.0 (4)	36.1 (13)	34.7 (17)	31.1 (37)		
	Professional experience (Years) % (*n*)	(3–10)	(11–20)	(21–30)	(>30)		0.160	3.41 (1.09–10.69) *p* = 0.035
		20.0 (2)	11.1 (2)	34.3 (12)	37.5 (21)	31.1 (37)		
	Education level % (*n*)	Nursing degree	Master’s degree	S-FCN	PhD (HS)		0.125	
		24.3 (18)	36.4 (4)	46.7 (14)	16.7 (1)	30.6 (37)		
	Healthcare service % (*n*)	PHC	PCES				0.720	
		31.3 (35)	22.2 (2)			30.6 (37)		
Q9	Age (years) % (*n*)	(26–35)	(36–45)	(46–55)	(56–65)		0.003	
		46.2 (6)	23.8 (5)	50.0 (18)	71.4 (35)	53.8 (64)		
	Professional experience (Years) % (*n*)	(3–10)	(11–20)	(21–30)	(>30)		<0.001	6.14 (2.26–16.67) *p* = 0.000
		40.0 (4)	11.1 (2)	54.3 (19)	67.9 (38)	52.9 (63)		
	Education level % (*n*)	Nursing degree	Master’s degree	S-FCN	PhD (HS)		0.117	
		50.0 (37)	36.4 (4)	70.0 (21)	33.3 (2)	52.9 (64)		
	Healthcare service % (*n*)	PHC	PCES				0.304	
		54.5 (61)	33.3 (3)			52.9 (64)		

Q5: Do you know what to do in the event of accidental dislodgement of a PEG tube? Q6: Have you ever reinserted a PEG tube after accidental dislodgement of the previous one? Q7: Do you know how to manage a PEG tube obstruction? Q8: Have you ever replaced a PEG tube due to obstruction? Q9: Have you ever replaced a PEG tube because it was due for scheduled renewal? S-FCN: Specialist in Family and Community Nursing; PhD (HS): Doctorate in Health Sciences; PHC: Primary Healthcare; PCES: Primary Care Emergency Service. Chi-square and Fisher’s exact tests were used for bivariate analyses. Odds ratios (OR) with 95% confidence intervals (CI) were derived from multivariable logistic regression for variables showing significant associations. Statistical significance was set at *p* < 0.05.

A total of 38.5% of nurses had received training, being more frequent among older nurses compared to younger ones (*p* < 0.001) and those with more years of professional experience (*p* < 0.001). Notably, nurses with 3–10 years of experience had not received specific training on PEG tubes, and only 11% of those with 11–20 years of experience had undergone such training. Furthermore, 98.3% of nurses considered training on PEG tubes to be necessary, with differences observed based on their education level (*p* = 0.029) (Table 5).

A total of 52.1% of nurses had provided health education to PEG tube patients and their families, with significant differences based on age (*p* = 0.002) and professional experience (*p* < 0.001). This practice was more common among older and younger nurses, and less frequent among those in the intermediate age group of 36–45 years (Table 5). Logistic regression analyses indicated that greater professional experience markedly increased the likelihood of having received specific training on PEG tubes (Q11; OR = 12.15) and of having provided health education to PEG tube patients and their families (Q13; OR = 5.87).

**Table 5:** Responses to Questions 10 to 13.

						Total	*p*	OR (95% CI) *p*
Q10	Age (years) % (*n*)	(26–35)	(36–45)	(46–55)	(56–65)		0.183	
		0.0 (0)	14.3 (3)	22.2 (8)	10.2 (5)	13.4 (16)		
	Professional experience (Years) % (*n*)	(3–10)	(11–20)	(21–30)	(>30)		0.060	
		0.0 (0)	5.6 (1)	25.7 (9)	10.7 (6)	13.4 (16)		
	Education level % (*n*)	Nursing degree	Master’s degree	S-FCN	PhD (HS)		0.741	
		14.9 (11)	9.1 (1)	13.3 (4)	0.0 (0)	13.2 (16)		
	Healthcare service % (*n*)	PHC	PCES				1.000	
		13.4 (15)	11.1 (1)			13.2 (16)		
Q11	Age (years) % (*n*)	(26–35)	(36–45)	(46–55)	(56–65)		<0.001	
		0.0 (0)	19.0 (4)	40.0 (14)	56.3 (27)	38.5 (45)		
	Professional experience (Years) % (*n*)	(3–10)	(11–20)	(21–30)	(>30)		<0.001	12.15 (2.71–54.30) *p* = 0.001
		0.0 (0)	11.1 (2)	41.2 (14)	52.7 (29)	38.5 (45)		
	Education level % (*n*)	Nursing degree	Master’s degree	S-FCN	PhD (HS)		0.169	
		32.9 (24)	27.3 (3)	55.2 (16)	33.3 (2)	37.8 (45)		
	Healthcare service % (*n*)	PHC	PCES				0.151	
		40.0 (44)	11.1 (1)			37.8 (45)		
Q12	Age (years) % (*n*)	(26–35)	(36–45)	(46–55)	(56–65)		0.830	
		100.0 (13)	100.0 (21)	97.2 (35)	98.0 (48)	98.3(117)		
	Professional experience (Years) % (*n*)	(3–10)	(11–20)	(21–30)	(>30)		0.181	
		100.0 (10)	100.0 (18)	94.3 (33)	100.0 (56)	98.3(117)		
	Education level % (*n*)	Nursing degree	Master’s degree	S-FCN	PhD (HS)		0.029	
		98.6 (73)	100.0 (11)	100.0 (30)	83.3 (5)	98.3 (119)		
	Healthcare service % (*n*)	PHC	PCES				1.000	
		98.2 (110)	100.0 (9)			98.3 (119)		
Q13	Age (years) % (*n*)	(26–35)	(36–45)	(46–55)	(56–65)		0.002	
		30.8 (4)	23.8 (5)	52.8 (19)	69.4 (34)	52.1 (62)		
	Professional experience (Years) %(*n*)	(3–10)	(11–20)	(21–30)	(>30)		<0.001	5.86 (2.16–15.89) *p* = 0.001
		30.0 (3)	16.7 (3)	48.6 (17)	69.6 (39)	52.1 (62)		
	Education level % (*n*)	Nursing degree	Master’s degree	S-FCN	PhD (HS)		0.451	
		45.9 (34)	54.5 (6)	63.3 (19)	50.0 (3)	51.2 (62)		
	Healthcare service % (*n*)	PHC	PCES				0.090	
		53.6 (60)	22.2 (2)			51.2 (62)		

Q10: Have you ever received an order to replace a PEG tube? Q11.Have you received specific training on PEG tubes? Q12. Do you consider PEG tube training necessary? Q13. Have you provided health education to PEG tube patients and their families? S-FCN: Specialist in Family and Community Nursing; PhD (HS): Doctorate in Health Sciences; PHC: Primary Healthcare; PCES: Primary Care Emergency Service. Chi-square and Fisher’s exact tests were used for bivariate analyses. Odds ratios (OR) with 95% confidence intervals (CI) were derived from multivariable logistic regression for variables showing significant associations Statistical significance was set at *p* < 0.05.

In the logistic regression analysis, the variables “being a Family and Community Nursing specialist” and “years of professional experience” showed a significant association with the dependent variable “currently caring for a patient with a PEG tube” (Table 6).

**Table 6:** Odd Ratios for Question 1 in multivariate regression.

	Q1: Do You Currently Care for Any Patients with PEG Tubes?		
Variables	OR	95%CI	*p*
S-FCN (yes vs. no)	6.495	2.414–17.480	0.000
Professional Experience (Years)	3.947	1.357–11.479	0.012

S-FCN: Specialist in Family and Community Nursing. Multivariable logistic regression analysis. OR = odds ratio; CI = confidence interval. Goodness of fit assessed using the Hosmer–Lemeshow test: χ^2^ = 0.595, *p* = 0.743.

Table 7 summarises the nurses’ responses to the open-ended question regarding their level of knowledge on caring for patients with PEG tubes. The responses were grouped into three categories: (a) Need for training, (b) Referral to hospital or Case Manager Nurse (EGC), and (c) Demand for updated guidelines, protocols, and directives.

## 4. Discussion

As highlighted, nearly all nurses who participated in this study had provided care to a patient with a PEG tube at some point, and half of them were actively caring for PEG tube patients at the time of the study. This reflects the frequent occurrence of this type of care, driven by factors such as population ageing and improved therapeutic outcomes in conditions that impair oral feeding. Logistic regression analysis confirmed that both specialization in Family and Community Nursing and greater professional experience significantly increased the likelihood of currently caring for PEG patients, highlighting the role of clinical expertise.

Moreover, two-thirds of the nurses had attended to patients with PEG tubes who presented some form of complication. The most frequently managed complication was accidental dislodgement, followed by obstruction and infection. These findings are consistent with data reported by other authors [15].

Tube dislodgement is a common reason for emergency department visits, reported in over 12.8% of patients with PEG tubes [16,17]. This dislodgement can occur due to deflation of the internal balloon or accidental removal, particularly in patients with cognitive impairment. In our study, half of the nurses reported knowing how to manage an accidental dislodgement of a PEG tube, with significant differences based on their health service (PHC: 59.8% vs. PCES: 22.2%; *p* = 0.038). Additionally, half of the participants had reinserted a PEG tube following accidental dislodgement, with this practice being significantly associated with older age (*p* < 0.001) and greater professional experience (*p* < 0.001). Notably, among nurses with less professional experience, only 10% of those with 3–10 years and 16% of those with 11–20 years of experience had reinserted a PEG tube under these circumstances.

In this regard, if the tube dislodgement occurs within the first month after placement, while the stoma is still immature, blind reinsertion of a new tube is not recommended due to the risk of positioning it in the peritoneal cavity. In such cases, the patient should be hospitalised, placed on a nil per os (NPO) regimen, and the replacement should be performed under endoscopic or radiological guidance. Conversely, if dislodgement occurs in patients with a mature stoma, a new tube can be inserted without the need for endoscopy. Temporarily, a Foley catheter (16–18F) may be used to maintain tract patency until a PEG tube is available [3,4,17].

Knowledge of these management protocols and ongoing updated training are essential for providing optimal care to patients with PEG tubes. Equally important is ensuring that nurses have access to the necessary material and human resources to effectively address these care demands.

PEG tube obstruction is also common, with an incidence of 23–35% among patients with these devices. Prevention is the key factor, but when obstruction occurs, the first step is to flush the tube with 50–60 mL of warm water or diluted pancreatic enzymes. If these measures are unsuccessful, tube replacement may be required [18].

In our study, two-thirds of the nurses stated that they knew how to manage a PEG tube obstruction, and this response was significantly associated with older age (*p* = 0.012) and greater professional experience (*p* = 0.003).

Regarding the incidence of stoma infection, it is estimated to occur in 5–25% of PEG tube placement procedures according to various studies, with rates decreasing to 3% when prophylactic antibiotic therapy is administered [19]. Clinically, stoma infection is characterised by erythema, induration, and purulent peristomal exudate. Proper stoma care is essential for preventing infections over the medium and long-term.

With respect to the replacement of the PEG tube at its scheduled renewal date, half of the study participants reported having performed this procedure. This response was also associated with age, being more common among older nurses (*p* = 0.003) and those with greater professional experience (*p* < 0.001). The average lifespan of a PEG tube is estimated to be around six months, although this period can extend to 1–2 years [4,18]. The first scheduled replacement of a PEG tube is recommended to take place in a hospital setting, either percutaneously or endoscopically. Subsequent replacements can be performed by home hospitalisation services or well-trained primary care staff, reducing both costs and patient transfers [4,20].

The association between professional experience and the ability to manage PEG tubes observed in this study suggests that practical exposure plays a decisive role in developing procedural competence. Nurses with longer professional trajectories were more likely to perform key procedures such as reinsertion, replacement, and obstruction management, reflecting how experiential learning and accumulated clinical judgment contribute to greater autonomy and confidence in handling these devices in primary care settings.

Regarding training, only 38.5% of participants reported having received specific education on PEG management, mainly older nurses and those with more years of professional experience (*p* < 0.001). In contrast, none of the nurses with 3–10 years of experience and only 11% of those with 11–20 years had received such training. Logistic regression analysis confirmed this association, showing that professional experience significantly increased the likelihood of both having received PEG-related training and having provided health education to patients and families. Moreover, 98.3% of nurses expressed the need for ongoing educational opportunities in this area, underscoring the gap between clinical practice and formal preparation.

In relation to the health education provided by nurses to patients with PEG tubes and their caregivers, our study found that only half of the nurses reported having offered such education, with differences according to age (*p* = 0.002) and professional experience (*p* < 0.001). This practice was more common among the oldest and youngest nurses and less frequent in the intermediate age group (36–45 years).

It has been observed that caregivers of individuals with PEG tubes often encounter problems after hospital discharge, including difficulties related to the stoma, tube care, or feeding administration. To improve the quality of life of both patients and their families, it is essential to provide them with appropriate education and training [10]. In this sense, education aimed at patients and caregivers can improve clinical outcomes, increase satisfaction for both, prevent complications, and reduce healthcare costs [21].

With respect to the responses to the open-ended question, nurses reported a lack of knowledge that generates professional insecurity, which could lead to unnecessary hospital referrals [15]. The feelings of fear and insecurity expressed by the nurses are consistent with the quantitative findings, which showed that only 38.5% of participants had received specific training on PEG management. This correspondence highlights how insufficient educational preparation can directly contribute to professional apprehension and uncertainty in managing PEG-related care.

Moreover, the qualitative references to frequent referrals to case manager or liaison nurses align with the low proportion of nurses who had performed key PEG-related procedures: only 31.1% had replaced a PEG tube due to obstruction (Q8), 48.7% had reinserted a dislodged tube (Q6), and only 13.4% had ever been instructed to replace one (Q10). These data suggest a pattern of dependence on specialised professionals, reflecting both the structural and organisational consequences of limited practical exposure to these procedures. Taken together, these findings underscore the need for structured training programmes that not only enhance technical competence but also foster autonomy and confidence among primary care nurses in PEG management.

On the other hand, and consistent with the perceived need for further training in PEG tube management, nurses particularly valued practical and applied training formats, such as mandatory seminars, regular clinical sessions led by specialist nurses, and clear visual protocols to identify and manage the most common complications [22]. Other studies have also indicated that home-based care and telephone counselling services are valuable resources to support patient follow-up [21].

Therefore, as described, following the placement of a percutaneous endoscopic gastrostomy (PEG) tube, various complications may occur—generally minor but potentially leading to morbidity, affecting patients’ quality of life, interrupting nutritional treatment, and increasing healthcare costs. For this reason, systematic long-term follow-up of these patients is recommended [18]. Primary care nurses play a key role in the prevention, reduction, and management of PEG-related complications, and their practice should be grounded in the most up-to-date evidence.

Accordingly, based on our findings and previous studies, it would be beneficial for primary care nurses to have access to clear instructions to provide to patients and caregivers, as well as simple educational materials to facilitate the education of both groups [17].

Therefore, as shown by our results and in line with previous studies, training related to PEG care requires more specific attention [21]. Moreover, the training content should align with current international guidelines, and simulation-based teaching methods could help prevent complications and serve as a practical educational resource [22].

This study has both strengths and limitations. Among its strengths, the questionnaire was designed by nurses who regularly care for these patients, ensuring familiarity with critical points of interest. In addition, responses were collected from nearly all provinces in Andalusia. However, several limitations should be noted. The sample size could be considered a constraint, as a larger sample might have provided greater statistical power and broader representativeness. The voluntary nature of participation may also have introduced a self-selection bias, since nurses who were more interested or motivated in the topic might have been more likely to respond. Moreover, the inclusion of nurses from other regions would have provided a broader national perspective. Finally, the questionnaire, although pre-tested for clarity, was not formally validated for reliability and validity. Future research should address this by developing and validating standardized instruments for PEG-related nursing care.

## 5. Conclusions

The findings of this study reveal that primary care nurses face complications associated with PEG tube patients without access to evidence-based guidelines, updated protocols, or directives, while simultaneously expressing a strong need for continuous training.

We believe that these consensus documents should be effectively disseminated to ensure their availability across various primary care settings. Furthermore, nutritional support units or home hospitalisation services could play a key role in improving care by offering practical and regular training programmes focused on PEG tube management.

Based on our qualitative findings, it is recommended that training programmes for primary care nurses combine practical seminars, online simulation-based modules, and scheduled clinical sessions led by experienced colleagues to ensure both accessibility and applicability. Additionally, clear visual protocols for the management of common PEG-related complications should be developed and disseminated across primary care services to standardise practice and enhance patient safety.

## Figures and Tables

**Table 1:** Questionnaire Questions.

**Demographic and professional background**	Age, gender, highest educational qualification, years of experience, province, zone, and work setting.
**Knowledge and experience with PEG tubes**	Q1 Do you currently care for any patients with PEG tubes? Q2 Have you ever cared for a patient with a PEG tube? Q3 Have you been called upon to address a PEG tube complication? Q4 Which of the following complications have you most frequently encountered? (Accidental dislodgement, obstruction, infection). Q5 Do you know what to do in the event of accidental dislodgement of a PEG tube? Q6 Have you ever reinserted a PEG tube after accidental dislodgement of the previous one? Q7 Do you know how to manage a PEG tube obstruction? Q8 Have you ever replaced a PEG tube due to obstruction? Q9 Have you ever replaced a PEG tube because it was due for scheduled renewal? Q10 Have you ever received an order to replace a PEG tube?
**Training received and perception of training needs**	Q11 Have you received specific training on PEG tubes? Q12 Do you consider PEG tube training necessary?
**Training provided to patients and families**	Q13 Have you provided health education to PEG tube patients and their families?
**Personal views on PEG tube management in Primary Care**	Q14 Please share any comments or observations about the level of knowledge of primary care nurses regarding the management of PEG tubes, including any recommendations or areas for improvement.

**Table 2:** Characteristics of Participants.

**Variable**	**% (*n*)**
Age (years)	
26–35	10.7 (13)
36–45	17.2 (21)
46–55	29.5 (36)
56–65	40.2 (49)
**Sex**	
Woman	67.2 (82)
Education level	
Nursing degree	60.7 (74)
Master’s degree	9.0 (11)
S-FCN	24.6 (30)
PhD (HS)	4.9 (6)
Professional experience (Years)	
3–10	8.2 (10)
11–20	14.8 (18)
21–30	28.7 (35)
More than 30	45.9 (56)
Healthcare service	
PHC	91.8 (12)
PCES	7.4 (9)
Geographical area (number of inhabitants)	
Urban (≥50,000)	59.0 (72)
Intermediate (5000 to 49,999)	29.5 (36)
Rural (≤4999)	7.4 (9)

S-FCN: Specialist in Family and Community Nursing; PhD (HS): Doctorate in Health Sciences; PHC: Primary Healthcare; PCES: Primary Care Emergency Service.

**Table 7:** Responses to question 14.

**1. Importance of training and continuous education**	I believe training is crucial not only for handling complications but also for proper care and placement. Continuous education is necessary for primary care professionals. Specific training on the care and management of PEG tubes is essential. It is always beneficial to refresh and share knowledge about PEG tubes or any new technique or device introduced into patient care. I think it is necessary to update knowledge periodically. There is an increasing number of patients with PEG tubes, so more training is needed for both professionals and families. There is significant lack of knowledge about caring for patients with PEG tubes, which generates fear and insecurity. As a result, nurses often avoid attending to these patients, perpetuating the issue. Very few professionals have knowledge of PEG tube management in primary care. The little knowledge I have was gained by asking the hospital unit responsible for this area. All staff need to be trained and have a clear understanding of the care required for patients with PEG tubes, as well as for their families and caregivers. Training is necessary to handle any incidents that may arise. While not everyone can know everything, teams should ensure that collectively they are equipped to address all potential issues. In my unit, I am the only one who changes PEG tubes. This remains an unresolved issue in primary care. The lack of knowledge creates insecurity among professionals, often resulting in unnecessary hospital referrals. This has led us to take measures to overcome fear of a relatively simple procedure. Many colleagues in primary care and emergency services do not know how to perform PEG tube changes or manage complications. More training is required for professionals, especially nursing specialisation. We cannot be expected to excel in everything. I believe courses and workshops should be conducted in each health centre and/or clinical sessions. More training on the management and complications of PEG tubes is needed. In primary care, training and information about PEG tubes are scarce or non-existent, and there is a prevailing fear of dealing with them. I think training is fundamental. Sometimes, lack of knowledge prevents professionals from managing this device properly. There is no specific training, and professionals have to educate themselves out of professional curiosity and the need to meet user demands. Training is important and can be delivered through organised courses, clinical sessions, or self-directed preparation using relevant documentation. The level of knowledge is medium to low. It is essential to implement training aligned with the changing needs of the population, such as those associated with this device. Scheduled clinical sessions are necessary for reviewing care practices and complications, as well as for updates on new models. Frequent refresher training on topics like PEG tubes, tracheostomies, etc., is crucial. Transfers or changes in position highlight a general lack of training, as nurses often move from other services without sufficient preparation.
**2. Referred to**	**Hospital** In my health centre, patients are referred to the hospital for PEG tube changes performed by a specialist. **Case manager nurse (or Liaison Nurse)** I serve as a case manager nurse, and in my centre, I have provided training on the care of patients with PEG tubes. We have very few patients with PEG tubes, and typically, any changes or issues are handled by the case manager nurse or liaison nurse. As a result, the rest of the staff are not very familiar with procedures related to this type of tube. My knowledge on the subject comes from having temporarily taken on the role of a case manager nurse, during which I was trained by my colleague from another management unit in our city. Generally, other colleagues rely on case manager nurses when they encounter a patient with a PEG tube. We either teach them if they are willing to learn or simply take it on as a specific responsibility of the case manager nurse. In primary care, PEG tube replacements are performed by the Case Manager Nurse. We have very few patients with PEG tubes, and typically, any issues or tube changes are handled by the case manager nurse. As a result, the rest of the staff are not very familiar with procedures related to this type of tube. It is an important topic that is perhaps insufficiently addressed in primary care. This could be due to the small number of patients with PEG tubes and the fact that it is primarily managed by case manager nurses. However, I believe training on this topic is crucial. Continuity of care When you care for a patient, they become the primary motivation to learn. It would be beneficial, in cases of hospital discharge with a PEG, to schedule an appointment with the primary care nurse before the discharge.
**3. Need for guidelines**	It is not only important to know how to place and maintain the PEG tube but also to understand when to change it, when it can be done at home (and whether any prior changes need to be made in the endoscopy unit), and who to contact if any issues arise.

## Data Availability

The data presented in this study are available on request from the corresponding author due to ethical reasons.

## Abbreviations

The following abbreviations are used in this manuscript:

PCES	Primary Care Emergency Service
PEG	Percutaneous endoscopic gastrostomy
PhD (HS)	Doctorate in Health Sciences
PHC	Primary Healthcare
PHC	Primary Health Zone
S-FCN	Specialising in Family and Community Nursing
